# The structure of dimensions of psychopathology in normative and clinical samples: Applying causal discovery to MMPI-2-RF scales to investigate clustering of psychopathology spectra and *p*-factors

**DOI:** 10.3389/fpsyt.2022.1026900

**Published:** 2022-11-10

**Authors:** Robbert J. Langwerden, Paul T. Van der Heijden, Tom Claassen, Jan J. L. Derksen, Jos I. M. Egger

**Affiliations:** ^1^Donders Institute for Brain, Cognition and Behaviour, Radboud University Nijmegen, Nijmegen, Netherlands; ^2^Community-Based Research Institute, Florida International University, Miami, FL, United States; ^3^Behavioral Science Institute, Radboud University Nijmegen, Nijmegen, Netherlands; ^4^Centre for Adolescent Psychiatry, Reinier van Arkel Mental Health Institute, 's-Hertogenbosch, Netherlands; ^5^Institute for Computing and Information Sciences, Faculty of Science, Radboud University Nijmegen, Nijmegen, Netherlands; ^6^Faculty of Psychology and Educational Sciences, Department of Clinical and Life Span Psychology, Vrije Universiteit Brussels, Brussels, Belgium; ^7^Centers of Excellence in Neuropsychiatry, Vincent van Gogh Mental Health Institute, Venray, Netherlands; ^8^Stevig Specialized and Forensic Care for People With Intellectual Disabilities, Oostrom, Netherlands

**Keywords:** BCCD, causal discovery, *p*-factor, HiTOP, Minnesota Multiphasic Personality Inventory-2 Restructured Form

## Abstract

We applied a Bayesian Constraint-based Causal Discovery method (BCCD) to examine the hierarchical structure of the Minnesota Multiphasic Personality Inventory-2-Restructured Form (MMPI-2-RF) Restructured Clinical (RC) scales. Two different general psychopathology super spectra (*p*-factor) scales were extracted from (1) all RC scales and (2) all RC scales except the RCd (Demoralization) scale. These *p*-factor scales were included in separate models to investigate the structure of dimensions of psychopathology in a normative (*n* = 3,242) and clinical (*n* = 2,466) sample, as well as the combined normative/clinical sample (*N* = 5,708), by applying the BCCD algorithm to obtain a data-driven reconstruction of the internal hierarchical structure of the MMPI-2-RF. Research on the underlying structure of the MMPI-2-RF has clinical relevance as well as conceptual relevance in the context of the HiTOP model. Results demonstrated that the syndromes measured with the RC-scales—in presence of a *p*-factor—cluster into six spectra: internalizing, disinhibited-externalizing, antagonistic-externalizing, thought disorder, detachment, and somatoform. These results may support a super spectrum construct, as it was necessary for obtaining a bottom-up reconstruction of this six-spectrum structure. We found support for superiority of a broad super spectrum with additional variance over and above demoralization, as it resulted in the clearest structure (i.e., clustering of the RC scales). Furthermore, our results indicate independent support for the bifactor structure model of psychopathology.

## Introduction

### The MMPI-2-RF and HiTOP

Contemporary diagnostic models of psychopathology are increasingly focused on quantitative approaches, mostly using factor analysis, to identify dimensionally operationalized psychopathology constructs and models (1–3), which has been particularly prominent in the context of personality pathology (4, 5). To define psychopathology beyond the predominantly categorical DSM-5 classifications (6), several dimensional models have been developed that rely on factor analysis to describe syndromes and symptoms in broader underlying variables (i.e., spectra). The Hierarchical Taxonomy of Psychopathology [HiTOP (3, 7–9)] is currently the most influential quantitative dimensional model of psychopathology. The HiTOP consortium proposed this data-driven hierarchical model, which incorporates various dimensions of psychopathology, including—from lower to higher level—symptoms, syndromes, subfactors, spectra, and a super spectrum (i.e., a general psychopathology-factor, *p*). The assessment of the HiTOP dimensions in clinical practice is work in progress (10), but as stated by Sellbom et al., at this time the Minnesota Multiphasic Personality Inventory-2-Restructured Form and 3 [MMPI-2-RF and MMPI-3; (11, 12)] are the most adequate and encompassing instruments to measure and operationalize the HiTOP model (13, 14).

**Figure 1:**
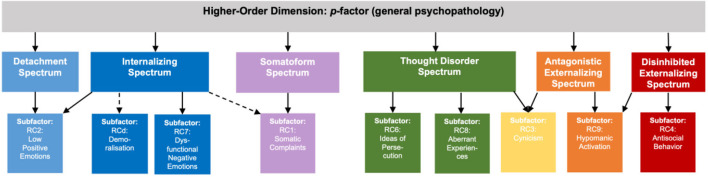
Hierarchical design structure of the MMPI-2-RF RC scales in accordance with the HiTOP model. Hierarchical design structure of the MMPI-2-RF, showing the six spectra and the corresponding RC-scales in accordance with HiTOP. Not depicted: the RC3 subfactor has associations with the RC2 subfactor (and Detachment spectrum) as well.

The MMPI-2-RF measures dimensional and hierarchically organized psychopathology constructs on various levels that map onto the HiTOP structure (3, 14). The hierarchical structure of the MMPI-2-RF includes three higher-order (H-O) scales, nine restructured clinical (RC) scales (15), twenty-three specific problem (SP) scales, and five pathological personality dimensions [i.e., PSY-5-r; (16)]. Of particular interest in this study are the nine RC scales (see Table 1), that resemble the subfactors in HiTOP (see Figure 1). From these constructs, the Demoralization scale (RCd) stands out, due to its construction and high clinical relevance. This construct formerly represented shared variance of the Clinical scales of the MMPI-2 and reflects general distress and general dissatisfaction with life. RCd is associated with the internalizing spectrum (11, 17) and correlates highly with suicidality and a range of depressive symptoms (18, 19). The other eight RC scales measure syndromes of somatic complaints (RC1), low positive emotions (RC2), cynicism (RC3), antisocial behavior (RC4), ideas of persecution (RC6), dysfunctional negative emotions (RC7), aberrant experiences (RC8), and hypomanic activation (RC9) (11, 15, 19). The MMPI-2-RF is one of the most utilized clinical assessment instruments globally. Further research on the dimensional structure of the MMPI-2-RF has clinical relevance as well as conceptual ramifications in the context of the HiTOP model (3, 14).

**Table 1:** Descriptives of RC-scales and H-O scales in the Combined Sample.

**Scale**	**Description**	***M***	***SD***	**α**
RCd	Demoralization	57.9	14	0.94
RC1	Somatic complaints	55	13.6	0.85
RC2	Low positive emotions	55.3	12.9	0.76
RC3	Cynicism	50.7	10.5	0.77
RC4	Antisocial behavior	56.1	14.1	0.79
RC6	Ideas of persecution	53.3	12.4	0.68
RC7	Dysfunctional negative emotions	55.2	12.8	0.86
RC8	Aberrant experiences	54.6	12.5	0.75
RC9	Hypomanic activation	52	11.5	0.77
EID	Emotional/internalizing dysfunction	57.4	14	0.93
THD	Thought dysfunction	53.5	12.4	0.73
BXD	Behavioral/externalizing dysfunction	54.4	13.9	0.79

T-scores and Cronbach's alphas obtained in the combined sample (*N* = 5,708).

Similar to the MMPI-2-RF structure, HiTOP is a hierarchy of clusters of syndromes and symptoms, grouping lower level symptoms under broadly defined spectra (3). The internalizing spectrum encompasses symptoms of negative affectivity, anxiety, fear, sexual problems, eating problems, and depressed mood (3, 20). A related somatoform spectrum is considered to encompass symptoms of pain disorder, illness anxiety symptoms, as well as other somatic symptoms (13, 14, 21). Two externalizing spectra encompass disinhibited-externalizing and antagonistic acting-out behaviors (14). A fifth spectrum thought disorder (or thought dysfunction) encompasses psychotic, schizotypal and paranoid features (21, 22). Finally, the sixth spectrum is labeled the detachment spectrum which is defined by low positive emotionality and avoidant/schizoid and dependent personality symptomatology (3, 23).

### HiTOP approach and advantages

Generally, the HiTOP model takes a quantitative, multidimensional, and hierarchical approach to the structure of psychopathology, which has several advantages over traditional, categorical classifications (3, 24). First, dimensional measures of psychopathology have evidenced superior reliability and validity to categorical diagnoses (25, 26). Also, dimensional profiles, such as those generated by the MMPI-2-RF, provide the opportunity for clinicians to reframe mental illness into strengths and vulnerabilities in various domains of functioning (24, 27). The hierarchical structure with broad dimensions at the head of the structure addresses the well-known problem of co-occurrence [i.e., comorbidity or symptom overlap; (28)] among diagnostic categories, whereas the small lower levels maintain the heterogeneity of symptoms and incorporate multifinality (7, 29). Hierarchical, dimensional measurement allows for comprehensive assessment of psychopathology; both adaptive and maladaptive personality dimensions (i.e., continua of personality, rather than clinical categories) fit well into the overall structure, given the numerous empirical links identified between personality traits and psychopathology (14, 30, 31). Finally, decreased use or disappearance of routinely assigned categorical, clinical “labels” may have a destigmatizing effect on the individual and their environment.

Despite these advantages, there are still several underexplained aspects of the HiTOP model, such as the lack of clarity about stability of the different levels of the model and the descriptive (as opposed to elucidative) nature of the model. In addition, the model is grounded in factor-analytic approaches, whereas several studies have indicated that psychopathology is better understood as networks of interconnected symptoms and traits (32–35). Moreover, some of the fundamental assumptions of HiTOP have recently been debated in the literature (8, 36). Nonetheless, the HiTOP structure has been studied across a variety of methods including self-report measures such as the MMPI (37–39), as well as interviews (40) and peer report studies (41). The dimensional nature of various types of psychopathology has been replicated across samples (42), cultures (21, 43, 44), and ages (45–47).

### General psychopathology factor

Cross-correlations between spectra may point toward a general psychopathology construct or *p*-factor, a broad factor that underlies various phenotypes of psychopathology (48) and is therefore positioned as a super spectrum in the HiTOP model (3). This super spectrum or *p*-factor construct has been compared to the *g*-factor [i.e., general mental ability; (49)] and has gained momentum using latent trait analysis and network analysis (50). Particularly, a high level of *p* may be an indicator of a general risk for various types of psychopathology. A genotype and phenotype *p*-factor has been replicated as a general dimension of psychopathology across ages, methods and countries (1, 51, 52). For a critical evaluation of the *p*-factor literature to date the reader is referred to Watts and colleagues (53).

The true nature, reliable measurement, and direct clinical relevance of this construct has not been fully determined (53). An important topic for the current study is the discussion about different statistical approaches in the *p*-factor literature [i.e., bifactor vs. hierarchical models (54)]. The bifactor model is hypothesized to explain spectral clustering of syndromes and symptoms only through the *p*-factor construct, while the hierarchical model poses that the *p*-factor predicts spectra which in turn predict the underlying syndromes and symptoms (55). Recent evidence points toward a bifactor model, in which the *p*-factor construct was primarily associated with impulsivity, impairment and neuroticism constructs (56).

### Replication of HiTOP's structure with *p*-factor

While most studies have evaluated the structural validity of the HiTOP model using traditional factor analytic methods, replicating the structure using advanced statistical methods without predefined assumptions about the structure would ensure the robustness and generalizability of the model (3, 57). Importantly, modern causal discovery methods are specifically designed for such hypothesis-free reconstructions by generating networks from data. Where classic methods of factor analysis compare different models by establishing a fit of the data and comparing likelihoods, causal discovery methods try to reconstruct the underlying generating network bottom-up in fully data driven approaches. With the ability to identify complex hierarchical networks in large data sets, they could provide a promising complementary tool for investigating the structure of the HiTOP model. Therefore, in the present study, we investigate the structure of the HiTOP model using a state-of-the-art causal discovery approach. We opt for the Bayesian Constraint-based Causal Discovery (BCCD) algorithm (58), because of its ability to handle latent confounders, and also provides an estimate of the reliability of each part of the network found. However, well-known alternatives like bootstrapped FCI (59) or HEJ (60) could also be applied. BCCD was previously used to successfully detect clustering patterns in the Wechsler Adult Intelligence Scale-Fourth Edition structure (61, 62).

**Figure 2:**
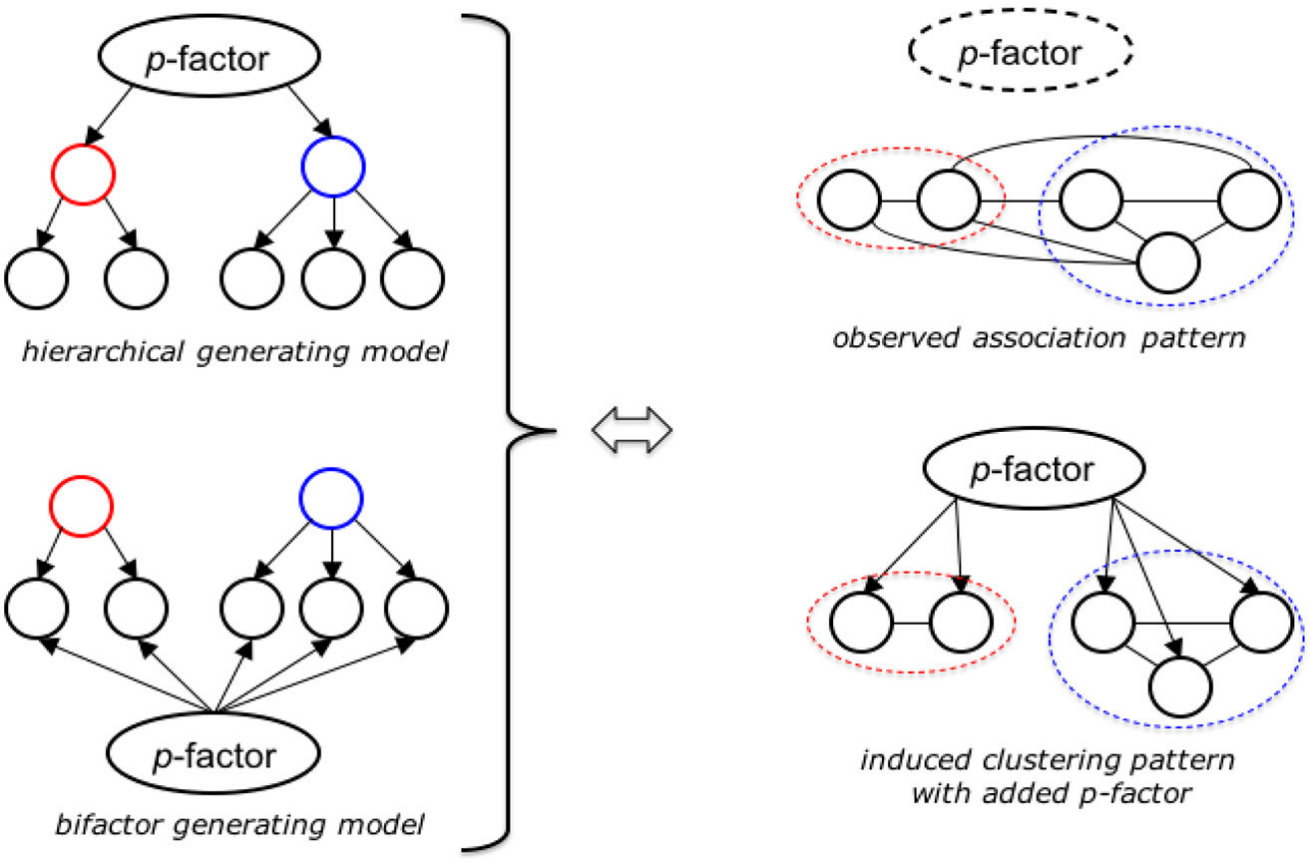
Explanatory models of the BCCD-method applied to dimensional MMPI-2-RF data. Higher order factor structure **(left)** revealed as clustering pattern through causal network analysis **(bottom, right)**, provided “true” *p*-factor is sufficiently approximated. Without adequate *p*-factor proxy the network shows a high degree of latent interactions between the observed factors (in our case the separate RC-scales).

The present study focuses on applying BCCD to MMPI-2-RF (11) RC scales (15) in both community and clinical samples. To investigate the existence, nature, and positioning of a general psychopathology factor, we extract two different *p*-factor scales from (1) all RC scale items and (2) all RC scale items except demoralization items. We examined the structure of the MMPI-2-RF scales in relation to these two operationalizations of the *p*-factor. The Demoralization construct was originally derived to capture a primarily-internalizing, overarching construct from the MMPI-2 Clinical scales. However, the *p*-factor is hypothesized to be associated not only with internalizing symptomatology but with all six spectra (i.e., internalizing, disinhibited-externalizing, antagonistic-externalizing, thought disorder, detachment, and somatoform) [e.g., (1, 56, 63)] and to be central at the top of the model as in a hierarchical model (14, 54, 55). Therefore, we compare the structure of both models with different *p*-factors (i.e., primarily defined by demoralization vs. broadly defined). If the HiTOP design *and* the hypothesized *p*-factors hold true, then we would expect clustering of RC-scales into six spectra (64). Through investigation of the cross links between subordinate dimensions of the model structures both with and without the general psychopathology dimensions, the present study can also shed new light on the discussion around bifactor vs. hierarchical structures of psychopathology [see Figure 2; (55)].

Work by Sellbom in particular has led to mapping of the RC scales onto the HiTOP model (13, 14), which has guided our hypotheses. We hypothesize clustering of the RC scales into 6 spectra, mapping onto the HiTOP spectral constructs, such that the constructs Demoralization (RCd) and Dysfunctional Negative Emotions (RC7) cluster into the internalizing spectrum. The detachment spectrum is proposed to be associated with Low Positive Emotions (RC2), which is also expected to show associations with the internalizing spectrum. The somatoform spectrum is in line with the construct measured by the Somatic Complaints construct (RC1). The disinhibited-externalizing spectrum is mapped onto the Antisocial Behavior construct (RC4), whereas the antagonistic-externalizing spectrum maps onto the Hypomanic Activation construct (RC9) and to a degree the Cynicism construct (RC3), the latter of which is expected to be associated with the thought disorder and internalizing spectrum as well (19). The sixth spectrum, thought disorder, is a clustering of the Ideas of Persecution (RC6) and Aberrant Experiences constructs (RC8) (13), with Cynicism (RC3) associations. We expect both *p*-factor scales (with and without demoralization) to further accentuate these existing syndrome clusters, with the *p*-factor scale without demoralization being the stronger overarching super spectrum by removing the most cross-variance between the clusters.

## Method

### Participants and sampling procedures

All participants completed the Dutch language version of the MMPI-2. Using the MMPI-2 booklet, MMPI-2-RF raw scores were generated, previously shown to be a valid method of conversion (11, 65). Combined gender norms T-scores were computed for all scales. For an overview of summary statistics of the MMPI-2-RF T-scores in the Combined sample, we refer to Table 1. All participants met the following validity inclusion criteria based on the Dutch language manual of the MMPI-2-RF: CNS < 14, VRIN-r/TRIN-r < = 80T, F-r < 120T, F-r < 100T, L-r < = 80T.

#### Normative subsample A

Participants in normative subsample A completed the MMPI-2 in 1992 in the context of standardization of the Dutch-language version of the MMPI-2 (66). This sample was stratified where needed to best represent normal distributions in the general Dutch population. All participants were financially compensated for their participation. MMPI-2 administration was computerized and participants who lacked the technical necessities were provided with the necessary equipment. For more information on the sampling procedures of normative subsample A, we refer to the manual of the first edition of the Dutch language version of the MMPI-2 (66). After removal of invalid participants and participants that also participated in the standardization of the Dutch language version of the MMPI-2 in 2012, the final 1992 sample included 1,157 participants (*M*_*age*_ = 43.6, *SD*_*age*_ = 14.3, 45.4% female).

#### Normative subsample B

Participants in normative subsample B were recruited in 2012 to update the norms of the Dutch language version of the MMPI-2. All participants were compensated for their participation, and administration was computerized (67). After removal of invalid participants, those who repeated assessments at both time points, and invalid survey responses, the final normative subsample B included 2,085 participants (*M*_*age*_ = 52.1, *SD*_*age*_ = 16.8, 48% female).

#### Normative sample

Normative subsamples A and B were merged into one Normative Sample for further analysis (*n* = 3,242, *M*_*age*_ = 49.1, *SD*_*age*_ = 16.4, 47.1% female).

#### Clinical sample

Independent from the normative sample administration, participants from six large, specialized inpatient and outpatient mental health facilities in the Netherlands filled out the MMPI-2 for the purpose of assessment and treatment planning. We removed participants with invalid survey responses, resulting in the final clinical sample (*n* = 2,466, *M*_*age*_ = 35.1, *SD*_*age*_ = 11.9, 42.3% female). These participants were included based on convenience sampling and inclusion followed their referral for psychological assessment and treatment planning because of multiple comorbid and severe mental disorders, including internalizing, externalizing and psychotic symptomatology.

#### Combined sample

The normative sample and clinical sample were merged into one Combined Sample (*N* = 5,708, *M*_*age*_ = 43, *SD*_*age*_ = 16.2, Range_age_ = 18–90, 45% female).

#### Sampling comparison

We compared the normative and clinical samples on age and gender. First, comparing the Normative Sample (*M*_*age*_= 49.1, *SD*_*age*_ = 16.4) with the Clinical Sample (*M*_*age*_ = 35.1, *SD*_*age*_ = 11.9) on age, statistically significant differences between the means were found [*t*(5, 706) = 35.7, *p* < 0.001]. Second, Chi-square tests of independence were conducted to assess differences in gender between the two samples. A statistically significant difference [χ^2^(1, N = 5, 708) = 12.9, *p* < 0.001] was found between the Normative Sample (47.1% female) and Clinical Sample (42.3% female). We addressed these differences by running BCCD analyses with and without age and gender to assess whether the structure was consistent.

#### Informed consent

This study involves a unique secondary analysis of earlier published datasets (68–71). First, regarding the clinical sample, approval was granted by the Institutional Review Board of Reinier van Arkel Mental Health Institute [Date: April 2007/No.: ZO/AL/mg.04.2007; previously reported on by Van der Heijden et al. (69). Data were gathered for the purpose of psychological assessment and treatment planning. We extracted anonymous data from large datasets and this research was conducted in accordance with the guidelines for archival research from Reinier van Arkel Mental Health Institute based on Dutch law (i.c. WGBO art. 458). Second, for the normative sample, participants completed the MMPI-2 in context of the standardization process of the MMPI-2 which was done by Center Data in the Netherlands. Data collection was done entirely anonymously. Data from the Dutch normative sample were made available by the test publisher with consent by the participants [see (68)].

### Analysis procedures

#### *p*-factor scales construction

In order to extract two general psychopathology (*p*-factor) scales, we first factor analyzed all RC scale items (i.e., 192 items) and then all RC scales items except items from RCd (i.e., 168 items) in the combined sample. We applied factor extraction on the combined sample (*N* = 5,708) to maximize the sample size and variability in constructing these scales. We ran exploratory factor analyses (EFAs) in SPSS version 28 (72) on both item pools (i.e., 192 and 168 items), fixing the factor extraction to 1 factor, with Maximum Likelihood extraction and 25 iterations. We extracted the 21 single highest loading items from the 192 and 168 item pools to construct two scales, as this number is the average number of items of the RC-scales.

#### Causal model analysis

We applied the Bayesian Constraint-based Causal Discovery algorithm (BCCD) on the available data to determine the hierarchical structure between the RC-scales, both with and without the two *p*-factor proxies. We only report models constructed using the combined sample data. We assessed five models (1) without *p*-factors and without RCd to assess free clustering of the RC scales, (2) with the *p*-factor that was based on eight RC scales, and without the Demoralization scale, (3) with the *p*-factor that was based on eight RC scales, and with the Demoralization scale, (4) with the *p*-factor based on all RC scale items (including RCd) with eight RC scales (not RCd), and (5) with the *p*-factor based on eight RC scales (without demoralization items) with nine RC scales (including RCd) as well as the three H-O scales to assess bifactor vs. hierarchical models. We reran these models for the different subsamples (normative/clinical) and with and without age and gender in the model.

Constraint-based causal discovery methods try to infer invariant features of the true underlying generating system from statistical patterns—conditional in- and dependence constraints—in the data. Under certain reasonable assumptions [Causal Markov and Causal Faithfulness, see (59)], in a principled way these can be combined into a single coherent causal model. The output consists of a graphical model that depicts the inferred relations between the observed variables, where edges correspond to direct interactions, and arrowheads signify the direction of causal influence. In the current study, we were primarily interested in the direct interaction structure represented by the edges. We chose BCCD because it is accurate, robust, and able to recognize interactions due to unobserved (latent) confounders, while it is able also to test the reliability of individual parts of the output model that will help us assess to what extent the output model follows the HiTOP design. However, any valid causal discovery method that can handle latent variables can be used.

The main principle behind our approach is depicted in Figure 2. When one considers a system with an underlying generating structure like the one depicted in the top left-hand side of Figure 2, with a single overarching top variable (*p*-factor), that affects a small set of intermediate variables (higher order factors), that each in turn determine a number of leaf variables (the separate RC scales/clusters). If there are no other direct interactions in the system and all variables are observed, then a causal discovery algorithm will be able to fully reconstruct the underlying graph. The same holds true if the relations follow the bifactor model depicted in the bottom left-hand side. Unfortunately, even though this matches our postulated HiTOP structure, we cannot verify this directly because: (1) the true *p*-factor is not observed directly, and (2) the clustering of RC-scales into higher-order factors is part of our hypothesis.

When a causal discovery algorithm is run on the same system but *without p*-factor or higher-order factors present in the data, then the many associations that exist between scales in the same factor, as well as between the separate scales from different factors will appear as induced cross links in the overall model. This effect on the output is depicted in the top right of Figure 2. If our hypothesis is correct *and* the *p*-factors constructed *via* the procedure described above is a sufficiently adequate proxy for the truest *p*-factor, then adding that proxy into the model should resolve and/or significantly weaken many of the induced spurious links between scales from different factors, leading to the output depicted in the bottom right of Figure 2. Therefore, if we do find this model behavior in our causal model output on application to the HiTOP data we can likely take it as a confirmation of the validity of both the global *p*-factor hypothesis as well as the approximation procedure above.

Finally, if we find good independent confirmation of the HiTOP structure in combination with the *p*-factor as indicated above, then we can repeat our causal network analysis with the predefined higher-order factors included to infer the “natural” structure of the system as a whole. In particular, we can then expect to find a model that either resembles the traditional hierarchical model in the top left side of Figure 2, or one that is more closely aligned to the so-called bifactor model in the bottom left. Naturally, a preference for either is not yet definitive proof, but it should at least make for a compelling argument of the most likely explanation for the given data, as it is strictly based on identifiable structural properties of the underlying generating system.

As the approach is entirely data driven, observing this behavior in a large, high-quality data set will provide strong independent verification for both the design of the MMPI-2-RF (see Figure 1) as well as the existence and validity of the constructed hypothesized *p*-factor within the HiTOP model. For the BCCD, regressive scores on the *p*-factor were used as well as raw scores on all RC scales and H-O scales. We therefore input the full-item raw RC-scales, the two raw 21-item *p*-factor into the analyses, as well as the full-item raw H-O scales (in certain models). We also entered a construct indicating between clinical and normative samples, as well as age and gender, to identify structural differences in structure, if any. This follows the recently published Joint Causal Inference (JCI) framework for combining data from multiple studies into a single coherent causal model (73).

## Results

### *p*-factor scales construction

We identified two sets of 21 items, one for the *p*-factor with RCd items included (i.e., based on 192 items; hereafter referred to as *p*-factor_rcd) and one for the *p*-factor scale without RCd items (i.e., based on 168 items, hereafter referred to as *p*-factor). For an overview of the originating scales of the highest loading items including factor loadings, we refer to Table 2. The 21 items making up the *p*-factor_rcd scale were all items originating from the RCd scale, with the exception of one RC7 item. The 21 items making up the *p*-factor scale were items from 7 different RC scales, not representing RC3. The Chi square tests of model fit indicated significant results for both the *p*-factor_rcd [χ^2^ (df = 18, 144) = 141,535.73, *p* < 0.001] and *p*-factor [χ^2^ (df = 13, 860) = 114,221.44, *p* < 0.001], which may be due to overpowering.

**Table 2:** Originating scales and factor loadings for the *p*-factor scales based on the MMPI-2 booklet.

	***p*** **_factor_rcd**		***p*** **-factor**	
	**Scale**	**Factor loading**	**Scale**	**Factor loading**
1	RCd	(0.73)	RC7	0.61
2	RCd	0.72	RC8	0.53
3	RCd	0.7	RC7	0.52
4	RCd	0.7	RC7	0.51
5	RCd	0.69	RC7	0.51
6	RCd	0.67	RC1	(0.5)
7	RCd	0.67	RC7	0.5
8	RCd	0.65	RC7	0.49
9	RCd	0.65	RC4	0.48
10	RCd	(0.64)	RC2	(0.48)
11	RCd	0.63	RC8	0.48
12	RCd	0.63	RC8	0.47
13	RCd	0.63	RC2	(0.47)
14	RCd	0.61	RC8	0.46
15	RCd	0.61	RC1	0.46
16	RC7	0.6	RC9	0.46
17	RCd	0.6	RC7	0.45
18	RCd	0.59	RC6	0.44
19	RCd	0.59	RC1	0.44
20	RCd	0.58	RC7	0.44
21	RCd	0.58	RC1	0.43

All are based on the combined sample (*N* = 5,708). Loadings in parentheses are negative.

*p*-factor_rcd, *p*-factor scale with RCd items; *p*-factor, *p*-factor scale without RCd items.

Correlational results of the two *p*-factor scales with the RC scales and PSY-5-r scales are displayed in Table 3. These indicate significant correlations of varying strength in all three samples with nearly all RC-scales and PSY-5-r scales. However, it is important to note this may be inflated due to partial item overlap between the RC scales and the *p*-factor scales. The exceptions to the overwhelming significant correlations were the scales AGGR-r (measuring the maladaptive personality trait Aggressiveness), DISC-r (measuring the maladaptive personality trait Disconstraint) and INTR-r (measuring the maladaptive trait Introversion/Low Positive Emotionality), which did not significantly correlate with the *p*-factor scales in all subsamples. Reliability analysis indicated that the *p*-factor scale with demoralization items had good to excellent in the Combined sample (Cronbach's α = 0.94), Normative sample (Cronbach's α = 0.88), and Clinical sample (Cronbach's α = 0.91). The *p*-factor scale without demoralization items demonstrated acceptable to good internal consistency in the Combined sample (Cronbach's α = 0.87), Normative sample (Cronbach's α = 0.74), and Clinical sample (Cronbach's α = 0.82).

**Table 3:** Correlations with RC, H-O, and PSY-5-r scales and reliability statistics for the *p*-factor scales by sample and subsample.

	***p*** **_factor_rcd**			***p*** **-factor**		
	**Combined**	**Normative**	**Clinical**	**Combined**	**Normative**	**Clinical**
*p*_factor_rcd	–	–	–	0.86**	0.74**	0.78**
*p*-factor	0.86**	0.74**	0.78**	–	–	–
RCd	0.99**	0.99**	0.99**	0.85**	0.73**	0.76**
RC1	0.59**	0.48**	0.45**	0.68**	0.53**	0.63**
RC2	0.68**	0.44**	0.63**	0.55**	0.28**	0.44**
RC3	0.27**	0.28**	0.34**	0.36**	0.36**	0.46**
RC4	0.48**	0.27**	0.29**	0.58**	0.41**	0.44**
RC6	0.43**	0.33**	0.31**	0.54**	0.46**	0.48**
RC7	0.79**	0.71**	0.75**	0.84**	0.78**	0.82**
RC8	0.54**	0.36**	0.42**	0.71**	0.57**	0.67**
RC9	0.22**	0.15**	0.16**	0.4**	0.39**	0.4**
EID	0.95**	0.89**	0.93**	0.83**	0.71**	0.75**
THD	0.45**	0.32**	0.33**	0.6**	0.49**	0.56**
BXD	0.32**	0.12**	0.17**	0.45**	0.3**	0.35**
AGGR-r	(0.2)**	(0.22)**	(0.18)**	(0.02)	(0.002)	0.06**
PSYC-r	0.5**	0.37**	0.4**	0.63**	0.52**	0.6**
DISC-r	0.21**	0.03	0.08**	0.32**	0.16**	0.24**
NEGE-r	0.82**	0.73**	0.75**	0.81**	0.73**	0.75**
INTR-r	0.38**	0.19**	0.4**	0.25**	0.03	0.22**
α ^a^	0.94	0.88	0.91	0.87	0.74	0.82

Combined refers to the Combined Sample (*N* = 5,708), Normative refers to the Normative Sample (*n* = 3,242), Clinical refers to the Clinical Sample (*n* = 2,466). Correlations in parentheses are negative. ^**^Significant at the 0.01 level (two-tailed). ^a^Cronbach's alpha reliability of the p-factor scales based on the factor structure of 21 items.

*p*-factor_rcd, *p*-factor scale with RCd items; *p*-factor, *p*-factor scale without RCd items.

### BCCD structure analysis

**Figure 3:**
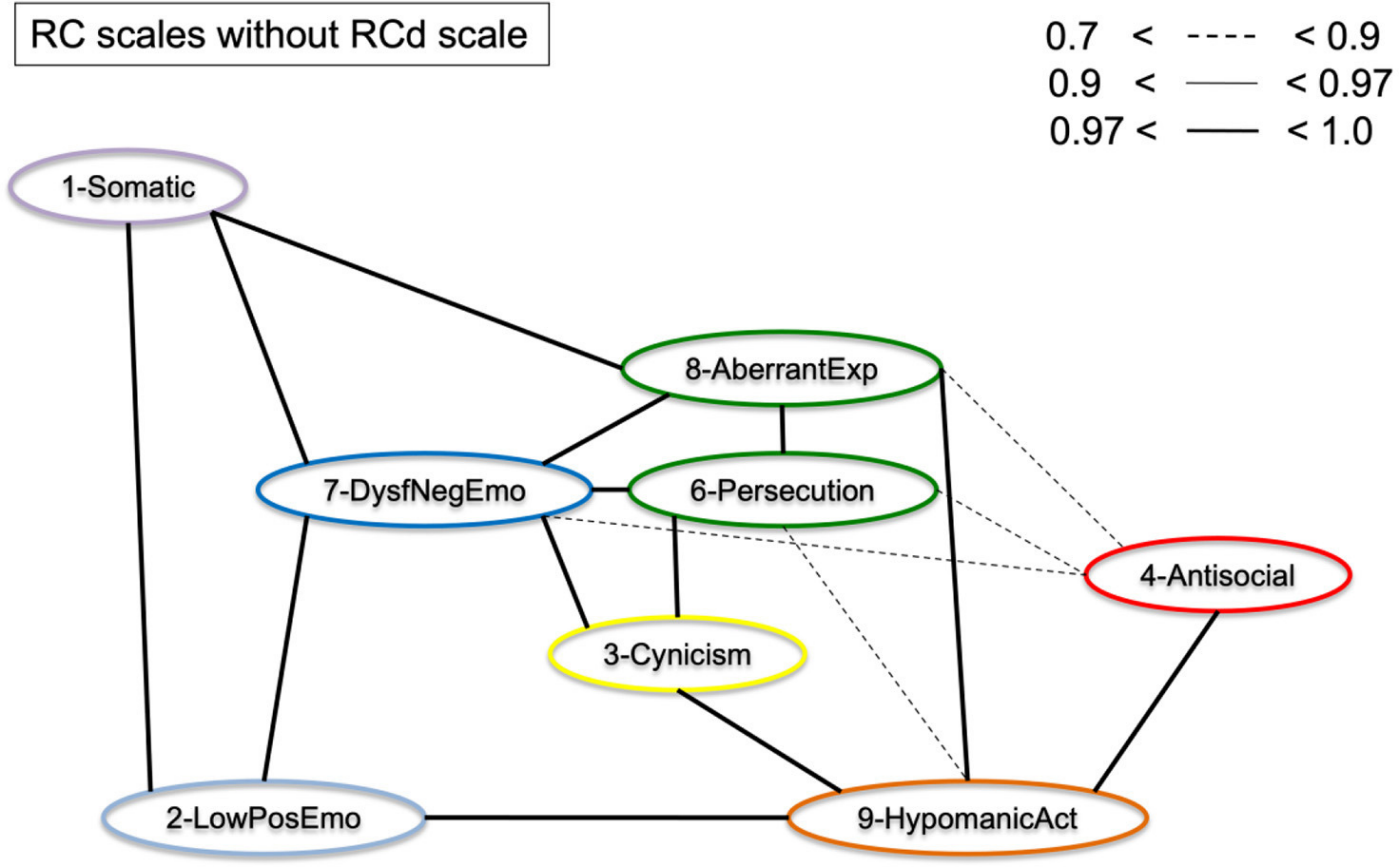
Graphical display of the structural organization of the RC scales without *p*-factor scales. Depicting RC-scales for the Combined Sample (*N* = 5,708).

**Figure 4:**
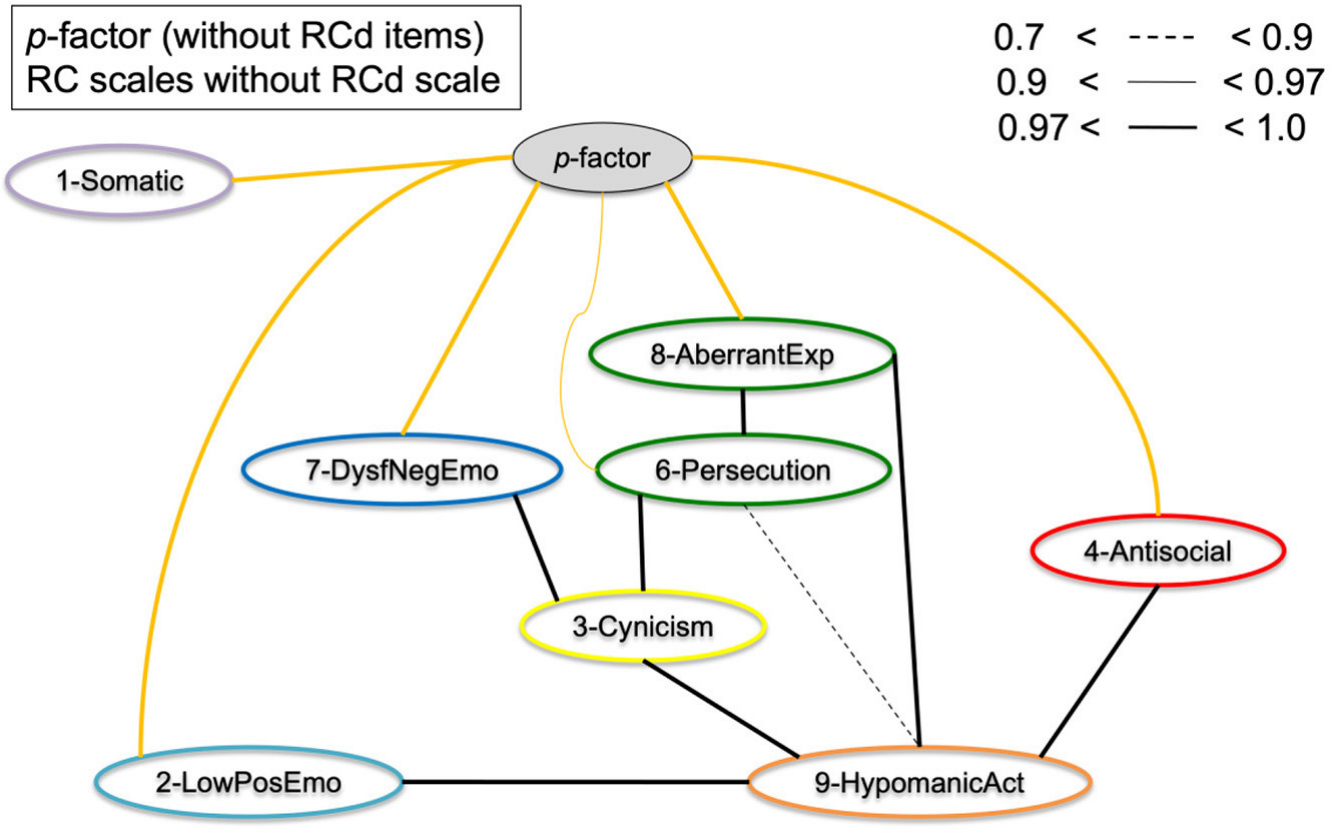
Graphical display of the structural organization of eight RC scales with *p*-factor without RCd items. Depicting RC-scales and *p*-factor scale without RCd items for the Combined Sample (*N* = 5,708). Yellow edges indicate *p*-factor links; black edges indicate cross-links between RC scales.

**Figure 5:**
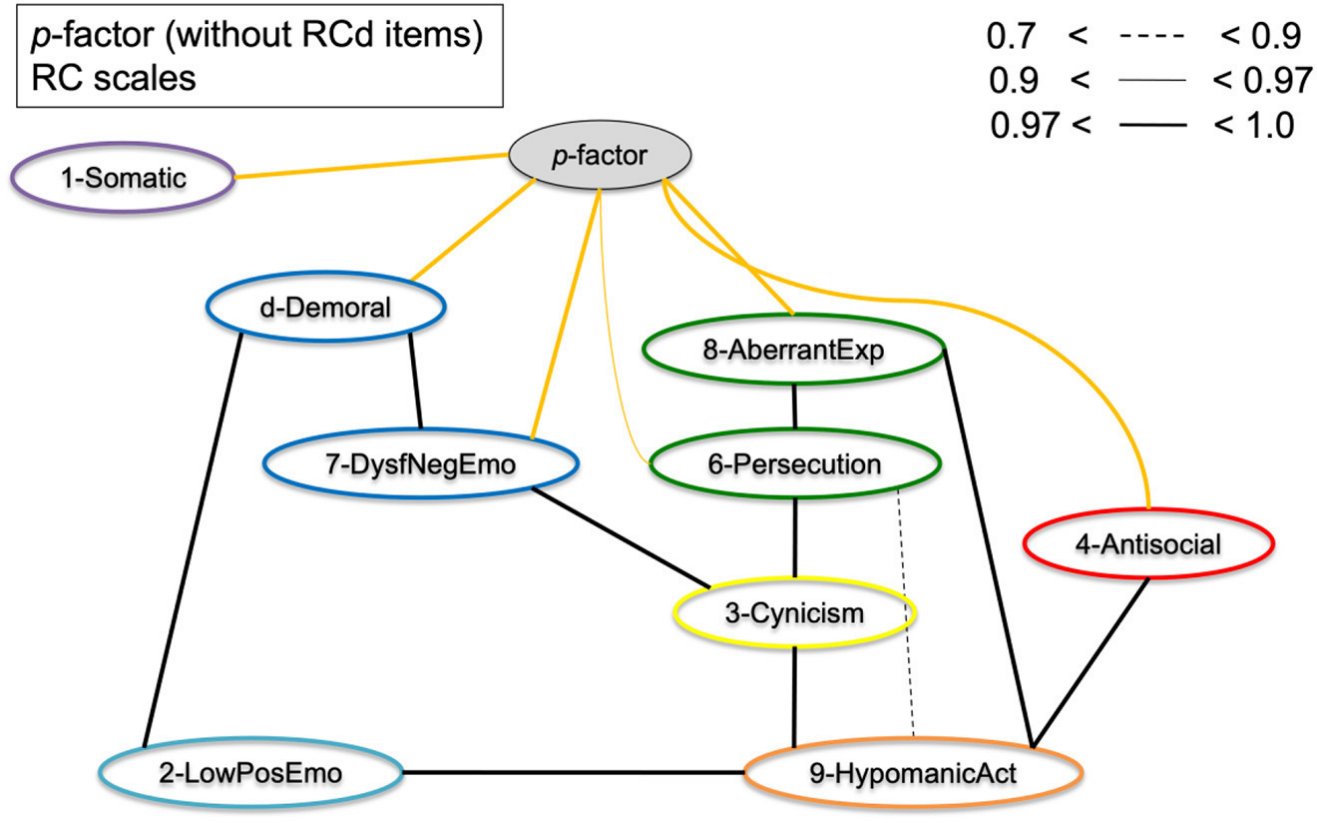
Graphical display of the structural organization of the nine RC scales (including RCd) with *p*-factor without RCd items. Depicting RC-scales and *p*-factor scale without RCd items for the Combined Sample (*N* = 5,708). Yellow edges indicate *p*-factor links; black edges indicate cross-links between RC scales.

**Figure 6:**
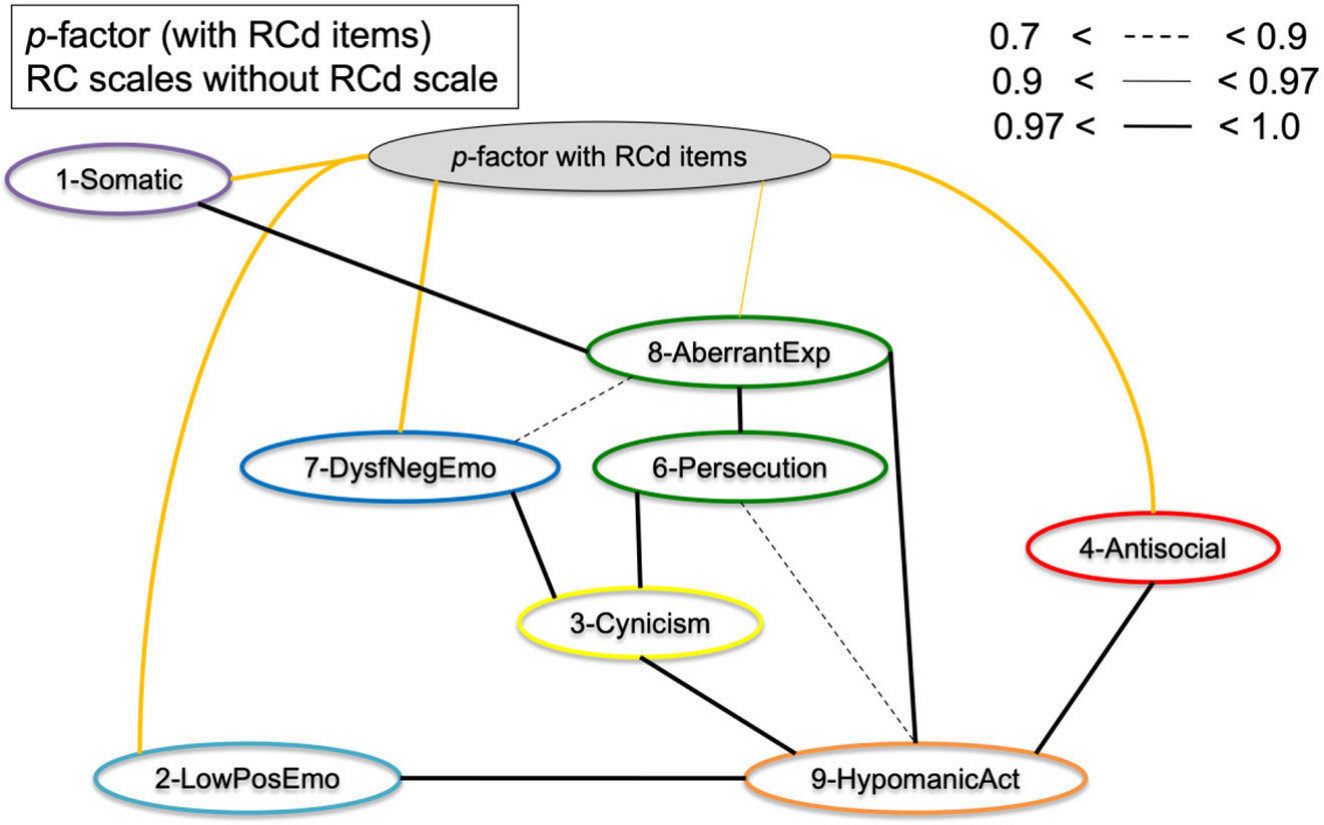
Graphical display of the structural organization of the eight RC scales with *p*-factor with RCd items. Depicting RC-scales and *p*-factor scale with RCd items for the Combined Sample (*N* = 5,708). Yellow edges indicate *p*-factor links; black edges indicate cross-links between RC scales.

**Figure 7:**
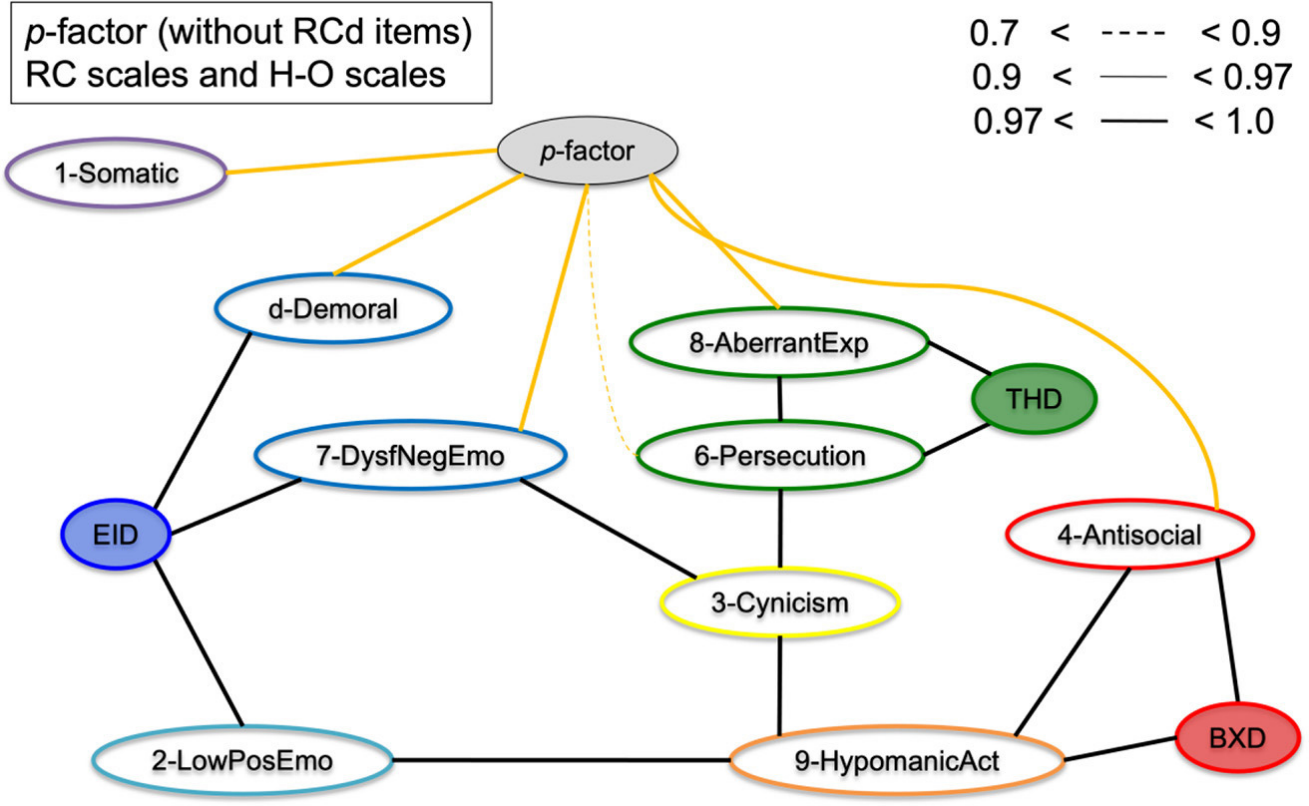
Graphical display of the structural organization of the nine RC scales, H-O scales, with *p*-factor without RCd items. Depicting RC-scales, H-O scales, and *p*-factor scale without RCd items for the Combined Sample (*N* = 5,708). Yellow edges indicate *p*-factor links; black edges indicate cross-links between RC and H-O scales.

Key BCCD model results are presented in Figures 3–**7**, which depict results in the combined sample (*N* = 5,708) only. For clarity reasons, we omitted any inferred causal directions in the output, as we are primarily interested in the emerging data driven clustering patterns. All original output data and figures are available upon request to the first author. Figure 3 displays clustering of the eight RC scales (excluding RCd) without adding either *p*-factor scale to the model. The results indicate clustering into the spectra as hypothesized, but at the same time show a number of cross-links between scales of different spectra. As a result, clustering patterns emerge, but with a high degree of overlap between scales and/or the presence of latent factors inducing spurious connections. In particular, the connections between RC1 and RC2, between RC7 and RC6, between RC7 and RC2 show this pattern of cross-linking between spectra.

Figure 4 displays the results of Figure 3, but with the *p*-factor scale without demoralization items added to the model. As can be observed from the graphical display, the pattern of clustering is more evident than without *p*-factor scale added to the model. Several cross-links, including between RC1 and RC2, between RC7 and RC6, and between RC7 and RC2 disappeared as a result. The *p*-factor scale without demoralization items captures variance between several RC scales to make the clusters more pronounced. The results show a more defined clustering of scales into the hypothesized six spectra (indicated by color): somatoform (purple; RC1), detachment (light blue; RC2), internalizing (dark blue, RC7), thought dysfunction (green: RC6 and RC8), disinhibited-externalizing (red: RC4), and antagonistic-externalizing [orange: RC9 and to a degree RC3 (yellow)]. Importantly, the *p*-factor scale (without demoralization items) is independently associated with these spectra, as visualized by the orange/yellow edges. Figure 5 illustrates the same results as Figure 4, but with the RCd scale added. As is evident from this model, the RCd scale positions within the internalizing and detachment clusters in the presence of the *p*-factor. This indicates the RCd scale is most strongly associated with these spectra.

Figure 6 shows the eight RC scales with the *p*-factor with demoralization items added to the model. Potentially due to this *p*-factor scale's close resemblance to the RCd scale (20 items out of 21 originate from RCd), the results indicate this *p*-factor is most strongly associated with the somatoform, internalizing, and detachment spectra. In this model, the RC1–RC8 link does not disappear, nor the association between RC7 and RC8. While the *p*-factor with RCd items behaves as an overarching *p*-factor scale to a degree, it mostly captures cross-variance in the internalizing/detachment side of the model.

To assess the bifactor vs. hierarchical underlying structures of the general psychopathology theories, Figure 7 shows the RC scales and Higher-Order scales with the *p*-factor without demoralization items. The results show that the *p*-factor is not associated with the Higher-Order constructs directly, but is associated with the RC level subfactors. This supports the bifactor model, as in a hierarchical model the *p*-factor would have directly associated with the Higher-Order scales.

Finally, these models were reconstructed for the clinical and normative samples independently, which evidenced only slightly different but robust structures similar to Figures 3–7. This indicates these findings are consistent across samples. In addition, we added age and gender to the model, which did not impact the structure of these models. For efficiency's sake, we have not depicted these models, but all models are available upon request.

Taken together, these results provide support for (1) clustering of subfactors (RC scales) into six spectra (all three samples), (2) the *p*-factor scale without demoralization items representing a general psychopathology factor due the fewest cross-links between the six spectra, (3) these findings being consistent across samples (normative/clinical) and with or without controlling for age and gender, and (4) the *p*-factor with demoralization items behaves itself as the RCd scale, which is as expected due to the high item overlap with the scale and one item from RC7 (internalizing spectrum).

## Discussion

We investigated the hierarchical structure of the MMPI-2-RF scales, using an underutilized Bayesian explorative approach. First, we extracted two scales as proxies of general psychopathology (*p*-factors), one with and one without demoralization items in the scale. This resulted in one scale with many demoralization items and one with items from 7 different RC scales represented. Next, we conducted a bottom-up constructed model, which resulted in six clusters (i.e., internalizing, disinhibited-externalizing, antagonistic-externalizing, thought disorder, detachment, and somatoform; see Figures 4, 5 in particular) mapping onto the spectra in the HiTOP-model (3, 14). The bottom-up clustering of the RC scale scores into spectra demonstrated a more pronounced structure after including the *p*-factor without demoralization items. These models are unique, as they are entirely data-driven and not dependent on the molding process of traditional factor analytic approaches (74, 75). We used a novel statistical approach in two large samples of both community and clinical origin.

Additionally, the results of the present study fit with the bifactor model as described in the literature (54, 55). As seen in comparing Figures 3, 4 as well as in Figure 7, the cross-links between RC-scales become less pronounced and connections between the spectra persist. This effect is evident when comparing models with and without *p*-factor: the spectra explain common variance between the RC-dimensions, indicating this to be a valid hierarchical level when constructing the dimensional structure bottom-up (54). Overall, our findings add to validity research of the MMPI-2-RF as a tool to hierarchically and multidimensionally operationalize the HiTOP.

### Hierarchical organization of the super spectrum

As hypothesized, the super spectrum without demoralization items was found to be an all-encompassing construct relative to spectrum-constructs in the Bayesian model, which emphasized the overarching nature of this construct. We constructed this *p*-factor scale without demoralization items, whereas the *p*-factor with demoralization items mostly resembled the RCd scale itself, despite inclusion of all RC items in the scale construction process. A single latent construct captured significant common variance among the symptomatology over and beyond the higher order factors, in line with earlier findings on the *p*-factor (1, 3). Theoretically it is of interest to investigate the “comorbid” patterns among individuals suffering from different forms of psychopathology, which appears to be demonstrated in at least six phenotypes of psychopathology according to these results (i.e., internalizing, disinhibited-externalizing, antagonistic-externalizing, thought disorder, detachment, and somatoform). Due to these spectrum-super spectrum relationships, the *p*-factor constructs should be seen in context of the larger model of hierarchical psychopathology dimensions, rather than as an autonomously meaningful construct (3, 53, 76). We found associations between the *p*-factor without demoralization construct and demoralization as a construct (Figure 5), despite the absence of item-overlap. However, when conducting these analyses with the *p*-factor with Demoralization items, the *p*-factor construct did not behave as an as overarching construct, which the *p*-factor without Demoralization items did. This may indicate the Demoralization construct is mostly associated with somatoform, internalizing (neuroticism), and detachment spectra, as was found in earlier research (19).

On an item-level, the *p*-factor without demoralization items seems to entail varying kinds of psychopathology, including emotional dysregulation, paranoid symptoms, various externalizing, and hypomanic symptoms (see Tables 2, 3). In the models with the *p*-factor without demoralization items, this scale functions as a broker-construct of the different spectra. Importantly, our findings indicate the model structure is much clearer in presence of a general *p*-factor without demoralization, which is in line with several previous studies (1, 77). We identified a clear tendency toward the bifactor model of psychopathology dimensions, rather than the standard hierarchical model (55), because second tier factors (higher-order factors) do not mediate between the *p*-factor and RC-scales, and appear disjoint from the *p*-factor when included in the full model.

### Hierarchical organization of the six spectra

The results in the present study replicate the hierarchical structure of dimensional measures of psychopathology, in line with the bifactor model (54). Importantly, in presence of a general psychopathology factor or super spectrum without demoralization items, 8 RC scales representing dimensional measures of psychopathology clustered into six spectra (21, 44, 78). These spectra map onto the six spectra described in the HiTOP model and are in line with previous research (14, 19).

First, Dysfunctional Negative Emotions (RC7) and—in some models—Demoralization (RCd), formed an internalizing spectrum, which is in line with previous factor analytic studies (20, 44). The Low Positive Emotions construct (RC2) could be seen as the detachment spectrum on its own since it did not associate with RC7 but directly with the *p*-factor (Figure 6), as well as with Hypomanic Activation (RC9). When adding the Demoralization construct, it did cluster into a more pronounced internalizing spectrum in the *p*-factor model, which may underline the demoralization component of the internalizing spectrum specifically. The Demoralization construct in this instance mediated between the internalizing spectrum (i.e., RC7) and the *p*-factor. Second, Antisocial Behavior (RC4) formed into a disinhibited-externalizing spectrum, whereas the Hypomanic Activation (RC9) scale and to a degree the Cynicism (RC3) scale formed an antagonistic-externalizing cluster. The two externalizing spectra were closely related; however, which replicates previous results indicating that instrumental acting-out and narcissistic, antagonistic types of externalizing symptomatology load on the same latent construct (70, 79). Further, Aberrant Experiences (RC8) and Ideas of Persecution (RC6) [and to a degree Cynicism (RC3)] clustered into a thought dysfunction spectrum, as is expected and in line with the literature (13). Finally, though somewhat associated to the internalizing spectrum, we found a somatoform spectrum, formed by Somatic Complaints [RC1, cf. (55)].

Taken together, these findings suggest a hierarchical factor structure on the spectrum level, not excluding the possibility of other spectra not measured in the present study, such as an obsessiveness subfactor or maladaptive trait (Anankastia). The utilization of BCCD as a probabilistic alternative to traditional factor-analytic approaches has allowed for independent validation of the three-factor model (33, 44), with the addition of a distinct somatoform spectrum, detachment spectrum, and splitting of the externalizing spectrum into antagonistic and disinhibited. With the publication of the new MMPI-3 (12), higher level constructs could be utilized as meaningful measures of psychopathology, potentially expanding on the already existing three higher-order scales of the MMPI-2-RF.

We discovered several anomalous yet interesting characteristics in the models. Cynicism (RC3) was diffusely positioned in relation to the spectra with overlapping associations with internalizing, thought dysfunction, and antagonistic-externalizing spectra. Cynicism as a construct does not refer to psychopathology in a narrow sense but encompasses other-referential beliefs. Despite clustering into the internalizing and thought dysfunction spectra respectively, Dysfunctional Negative Emotions (RC7) and Cynicism (RC3) were found to be associated in most models. This connection is presumed to be the result of conceptual overlap, or the co-occurrence of constructs measured by both scales that might be labeled as indignation or resentfulness, which captures both negative emotionality and paranoid tendencies (i.e., the feeling that one is treated unfairly) as both scales demonstrate moderate to strong relations with paranoid personality features (39, 70, 71). The latter may be due to the notion that the construct Cynicism (as captured in the MMPI-2-RF) seems to be a more diffuse amalgamation of overlapping aspects between the higher order factors, and persists as an extremely stable pattern, with or without the *p*-factor without demoralization items. Further, we found a strong relationship between Cynicism (RC3) and Hypomanic Activation (RC9), potentially resulting from common features regarding negative attitudes toward others, either due to narcissistic features or distrustful beliefs; and possibly underlying is self-referential mistrust (17). This is expressed in the antagonistic-externalizing spectrum. It is important to note that Cynicism (RC3) was not represented in the *p*-factor scale.

A robust association emerged between Low Positive Emotionality (RC2) and Hypomanic Activation (RC9). We suspect this to be a product of clinical overlap, including social disengagement and lack of energy—symptoms of Low Positive Emotionality (RC2), linked to introversion—and overactivity and restlessness due to hypomanic symptomatology, linked to extraversion (80). Moreover, Detachment can be formed by Low Positive Emotionality (RC2) by itself, which further explains this association (14). Low Positive Emotionality (RC2) is also associated with Aberrant Experiences (RC8), possibly due to psychotic depressive symptoms and negative symptoms (avolition) in the clinical sample. We found a weaker association between Ideas of Persecution (RC6) and Hypomanic Activation (RC9), both characteristics of bipolar disorder with psychotic features. In addition, paranoid ideas co-occur with narcissistic and antagonistic personality features (81). The aforementioned cross-spectrum relationships underline the notion that the spectra are not isolated dimensions, but rather that there are commonalities between key psychopathology dimensions on lower levels of the hierarchy (76).

### Clinical implications of the present findings

Our findings have several clinical implications. First, the outcomes support validity of the MMPI-2-RF scales, as well as the MMPI-2-RF's hierarchical structure of these measures. Second, latent constructs on a spectrum or super spectrum level were found in a combined clinical and normative sample, underlining the robustness of this hierarchical dimensional model of psychopathology. These dimensions could be targeted by spectrum-wide interventions. For example, the Unified Protocol for Emotional Disorders (82) may target the internalizing and detachment spectra, such as anxiety and mood symptomatology (i.e., “emotional disorders”). Targeting the common spectra in transdiagnostic treatment rather than having different sequential treatments targeting different forms of symptomatology seems not only be equally effective clinically [e.g., (83)], but also more cost-efficient for both the individual and for the health care system at large.

Second, as suggested by Caspi et al. (1) an elevated *p*-factor scale score may indicate a risk for several types of psychopathology for both the individual, but also for their family members. The relationship with Demoralization in our results may imply that high *p*-factor levels could indicate a profound mental illness risk, family members who are at risk, and lower quality of life as a result (1). Clinicians could keep these implications in mind if this construct were to be incorporated into diagnostic paradigms or manuals. Adequate measurement of a more developed *p*-factor construct may possibly be a meaningful addition to diagnostic assessment, particularly in light of transdiagnostic treatments targeting psychopathology overall (84). As opposed to the top-down development of general intelligence *g* and cognitive domains consecutively, the present study emphasizes the “bottom up” construction of a general psychopathology factor from underlying spectra. However, we urge clinicians not to reify the *p*-factor and not to view a potential general psychopathology scale as a stand-alone clinical tool, let alone the scales developed in the present study. As argued by several scholars [e.g., (3, 76)], the super spectrum is a part of a larger taxonomy of dimensions and should therefore not be regarded as clinically all-encompassing as such a single score may have a similar stigmatizing effect to multiple categorical labels.

### Limitations and future directions

The present study has several constraints that warrant noting. Regarding our statistical procedures, our main limitation is overlap between the items of the RC scales and the constructed *p*-factor scales. This may overestimate associations between the *p*-factor scales and different clinical constructs. Further, we were not able to cross-validate our *p*-factor measures with external measures, limiting the validity of these scales. The *p*-factor scales constructed were mere proxies and produced only with exploratory factor analysis. Additionally, we solely relied on the RC scales as measure of psychopathology, which—despite its well-established validity and reliability—may limit the generalizability of results. For example, compulsivity (i.e., the Anankastic domain) is not measured by the RC scales, despite this being a clinically relevant spectrum among other personality disorder domains described in the ICD-11 (85). Further research using all hierarchical levels, including the SP-scales, new MMPI-3 scales, and PSY-5-r scales, may elucidate the structure even more comprehensively [e.g., (13)]. Eating disorder symptomatology as well as sexual dysfunctions and disorders are not directly measured by the MMPI-2-RF, but may possible form other distinct spectra. Also, the MMPI is based on self-report to measure pathology, which can be seen as indirect and potentially biased.

We encourage future scientific efforts to be focused on further replication of the HiTOP model and dimensional measurement of psychopathology using novel methods, with a specific focus on bifactor vs. hierarchical nature of these models. While this wasn't the goal of the present study, more research would be needed to construct and validate a psychometrically sound *p*-factor scale and dimension, particularly given the finding that multicollinear scales will likely share a latent factor (53, 86). The benefits of the independent analytic approach based on causal model discovery could be brought to other applications or areas of research in psychopathology and beyond. Not because it is fundamentally “superior” than our existing tools, but because it can provide an alternative approach to available data that may offer new insights in the relevant underlying processes. The latter can be difficult to obtain in any other way. Alternatively, it could be an effective tool for independent confirmation of existing models. BCCD could play a role as an initial exploratory means of analysis for new data sets, as well as help formulate new hypotheses by virtue of an intuitive graphical representation of a system.

### Conclusions

Using a highly innovative, inferential statistical method, we produced an entirely data driven model in which constructs of psychopathology clustered into six spectra, replicating the HiTOP's main spectra in a combined normative and clinical sample. Moreover, *p-*factor proxies were constructed as super spectra (3), resulting in the finding that one *p*-factor (not representing Cynicism and Demoralization) may be underlying to six syndromic constructs of psychopathology.

## Data availability statement

The data analyzed in this study is subject to the following licenses/restrictions: the datasets are not publicly available. The corresponding author can be contacted for more information. Requests to access these datasets should be directed to RL, robbert.langwerden@donders.ru.nl.

## Ethics statement

The studies involving human participants were reviewed and approved by Institutional Review Board of Reinier van Arkel Mental Health Institute. The patients/participants provided their written informed consent to participate in this study.

## Author contributions

RL, PH, TC, and JE contributed to the study conception and design. Material preparation and data collection was performed by PH, JE, and JD. Data analyses were performed by RL, PH, and TC and were verified by the rest of the authors. The first draft of the manuscript was written by RL and TC. All authors commented on various versions of the manuscript. All authors read and approved the final manuscript.

## Conflict of interest

The authors declare that the research was conducted in the absence of any commercial or financial relationships that could be construed as a potential conflict of interest.

## Publisher's note

All claims expressed in this article are solely those of the authors and do not necessarily represent those of their affiliated organizations, or those of the publisher, the editors and the reviewers. Any product that may be evaluated in this article, or claim that may be made by its manufacturer, is not guaranteed or endorsed by the publisher.
